# Hair cortisol concentration in finishing pigs on commercial farms: variability between pigs, batches, and farms

**DOI:** 10.3389/fvets.2023.1298756

**Published:** 2024-01-22

**Authors:** Pierre Levallois, Mily Leblanc-Maridor, Anne Lehébel, Solenn Gavaud, Blandine Lieubeau, Julie Hervé, Christine Fourichon, Catherine Belloc

**Affiliations:** ^1^Oniris, INRAE, BIOEPAR, Nantes, France; ^2^Oniris, INRAE, IECM, Nantes, France

**Keywords:** hair cortisol, stress, biomarker, pig, welfare, health, field study

## Abstract

Hair cortisol is a stress indicator and could be used to assess the pigs’ exposure to stressors in the weeks/months prior to non-invasive hair sampling. The main aim of this study was to describe the hair cortisol concentration (HCC) variability between individuals within a batch, between farms and between batches within a farm. The secondary aim was to determine how the number of sampled pigs influences the characterization of HCC within a batch. Twenty farrow-to-finish pig farms were recruited considering the diversity of their management practices and health status (data collected). Hair was sampled in two separate batches, 8 months apart. The necks of 24 finishing pigs were clipped per batch the week prior to slaughter. To describe the variability in HCC, an analysis of the variance model was run with three explanatory variables (batch, farm and their interaction). To identify farm clusters, a principal component analysis followed by a hierarchical clustering was carried out with four active variables (means and standard deviations of the two batches per farm) and 17 supplementary variables (management practices, herd health data). We determined how the number of sampled pigs influenced the characterization of HCC within a batch by selecting subsamples of the results. HCC ranged from 0.4 to 121.6 pg/mg, with a mean of 25.9 ± 16.2 pg/mg. The variability in HCC was mainly explained by differences between pigs (57%), then between farms (24%), between batches within the same farm (16%) and between batches (3%). Three clusters of farms were identified: low homogeneous concentrations (*n* = 3 farms), heterogeneous concentrations with either higher (*n* = 7) or lower (*n* = 10) HCC in batch 2 than in batch 1. The diversity of management practices and health statuses allowed to discuss hypotheses explaining the HCC variations observed. We highlighted the need to sample more than 24 pigs to characterize HCC in a pig batch. HCC differences between batches on six farms suggest sampling pigs in more than one batch to describe the HCC at the farm level. HCC variations described here confirm the need to study its links with exposure of pigs to stressors.

## Introduction

Minimizing distress is essential to ensure the welfare of pigs on farms (1, 2). Distress is an aversive, negative state in which coping and adaptation processes fail to return an organism to physiological homeostasis (3). Distress is caused by the exposure of an individual to stressors. Stressors can be physiological or psychological threats, either real or perceived (4, 5).

On pig farms, stressors can be multiple and occur at various stages of life. It can be assumed that the exposure of pigs to stressors varies from farm to farm and from batch to batch within the same farm. For instance, the presence or absence of castration, tail docking or ear tagging from one farm to another could lead to different exposure of piglets to stressors (6).

The intensity of the stress response can vary from one animal to another when facing with the same stressor, depending in particular on their past experiences (7). Cortisol is a widely used indicator to assess the stress response in most mammals (8). Cortisol can be quantified in various matrices, including blood, saliva, urine, feces, and more recently in hair (8–10). Depending on the matrix, quantified cortisol can provide information about stress levels in different time windows prior to sampling. Serum or salivary cortisol provides information on stress levels at a specific moment, corresponding to a few minutes before sampling. Urinary or fecal cortisol (or its metabolites) reflects the stress level experienced during a period of accumulation, corresponding to several hours until urination or defecation occurs (10). Since cortisol accumulates during hair growth and remains *a priori* stored in the hair for several weeks or even months, hair cortisol is considered as a biomarker of chronic stress in humans (11, 12). In pigs, hair cortisol could potentially be used to assess stress throughout a long time period prior to sampling on commercial farms. Consequently, hair cortisol could possibly be a measure of on-farm pig welfare assessment. However, this possibility still needs to be assessed.

Measuring hair cortisol offers several advantages. Hair sampling is non-invasive, painless, and often does not require the animal to be restrained. Animal handling during hair sampling does not distort the measured concentration, as demonstrated by studies on dairy cows and reindeer (13, 14). Cortisol secreted during handling is unlikely to be present in hair samples, unlike blood samples. Hair samples can be stored at room temperature and protected from light for at least 1 year without significantly altering cortisol concentration, as studies on dairy cows, bears, and chimpanzees have shown (13, 15, 16).

Little is known about the mechanism of incorporation of cortisol into hair. Therefore, the definition of the time window considered by hair cortisol is uncertain (a few weeks or several months) (12). Cortisol could incorporate into hair through passive diffusion from blood (17). In that case, cortisol would incorporate into hair only during hair growth when hair follicle is linked to blood capillaries. Knowledge about the pig hair cycle on commercial farm is scarce. Watson and Moore (18) showed that hair growth is continuous but varies through time in piglets. Evidences showed that cortisol seems to accumulate in hair of growing pigs both before weaning and between weaning and slaughter (19). Therefore, hair cortisol measured at the end of the finishing period could potentially provide information on the exposure of pigs to stressors throughout their life on farm.

To our knowledge, studies providing results on hair cortisol in finishing pigs on commercial farms were carried out on a single farm (19–21). There is a lack of data to determine how hair cortisol concentration at the end of the finishing period varies in different non-experimental farming contexts. In absence of variation at the end of the finishing period, the hair cortisol concentration would provide only limited information regarding long-term stress. Therefore, it is important to describe the variability of hair cortisol concentration in finishing pigs before carrying out further research. It is assumed that hair cortisol concentration of finishing pigs may vary between farms and between batches within the same farm due to different exposure to stressors throughout their lives.

The number of animals sampled to describe hair cortisol concentration within a batch must consider the interindividual variability in stress response. Although little is known about interindividual variability in the literature, the study by Gavaud et al. (19) reported hair cortisol concentrations ranging from 30.0 to 177.6 pg/mg in 67 21-week-old pigs from three batches on the same conventional farm. The sample size must therefore be sufficient to take account of these interindividual differences.

Nevertheless, the feasibility of a hair cortisol measurement protocol is a limit to the determination of sample size, particularly when sampling is carried out on several farms. Total sample size in previous studies of hair cortisol at the end of the finishing period has ranged from 42 to 97 pigs, distributed over one to three batches within a single farm (19–23). Sampling several dozens of pigs per batch across several batches and farms may not be feasible given the constraints of laboratory protocol (e.g., labor time and cost of analysis). Consequently, to characterize the variability in hair cortisol concentration in finishing pigs, the sampling design must strike a balance between the number of farms and the number of pigs sampled per farm.

The aim of this exploratory study was to describe the variability in hair cortisol concentration in finishing pigs. Firstly, we wanted to characterize interindividual differences within a given batch. Secondly, we wanted to identify potential differences between farms (reflecting different levels of exposure to stressors). Thirdly, we wanted to identify potential differences between batches on the same farm. Finally, a secondary objective was to determine how the number of pigs sampled influences the characterization of hair cortisol concentration within a batch.

## Methods

### Description and recruitment of farms

This study was carried out as part of the European research project HealthyLivestock. A cohort of 20 farrow-to-finish pig farms in western France was monitored longitudinally. Farms were recruited based on criteria relating to the diversity in management practices and health status. This diversity was assumed to introduce variability in pig exposure to stressors across farms (in terms of nature, number, and intensity). The farms housed from 70 to 800 sows, with an average of 244 sows. Within the cohort, two farms were organic and 18 were conventional. Seven farms out of the 18 conventional had a specification: four farms were Label Rouge [product quality specification with minimal age at slaughter of 182 days and conditions for husbandry including lower animal densities, for details see (24)], two farms were antibiotic-free from birth, one farm was antibiotic-free from 42-day-old. Data were collected during two visits. During the initial visit, tailor-made health plans were formulated by veterinarians. The second visit was carried out on average 8 months later to assess the herd health evolution (25).

### Selection of sampled pigs

Pigs were sampled at the end of the finishing period at an average age of 165 ± 10 days. Sampling was carried out in two batches (one batch per visit), 8 months apart. Therefore, sampled pigs were different animals in the two batches on the same farm. The first sampling was performed in winter/early spring then the second one in autumn/early winter. Twenty-four pigs were sampled in each batch. Sample size was determined by reconciling the need for a sufficient sample size to describe the interindividual variability in hair cortisol concentration within a batch and the feasibility of the protocol. Sampled pigs were randomly selected within three or four pens, except on two farms where pigs were sampled in, respectively, one and two pens due to housing facilities. Pens were randomly selected on farm with more than four pens. Sex and breed of sampled pigs were not recorded. However, no variation of the hair cortisol concentration should be induced due to a sex effect for pigs aged of 165 ± 10 days (26). Most farms housed only castrated boars and females (Supplementary Table S1). Regarding breeds, all sows were Large White x Landrace and boars were predominantly Pietrain or sometimes Duroc. Our sample did not allow us to test a putative breed effect.

### Hair sampling procedure

Sampling was carried out without restraint by clipping the hair as close as possible to the skin of the dorsal area of the neck region, to sample the total length of the hair. Moreover, this method avoided to sample hair follicles likely to contain cortisol (via blood or their potential endocrine activity) (23, 27). The sampling region remained unchanged to avoid potential concentration variations between body regions (15, 16, 23). The neck was chosen as the sampling region to minimize potential contamination of hair with urinary or fecal cortisol (28, 29). At least 40 mg of hair were sampled per pig. The samples were placed in envelopes and stored in the dark at room temperature for a period ranging from 2 to 10 months until the measurements of hair cortisol concentration.

### Measurement of hair cortisol concentration

The protocol used to measure hair cortisol concentration was based on Gavaud et al. (19), adapted from Heimbürge et al. (26). Each hair sample was placed into a 15 mL polypropylene tube for two wash cycles. The first cycle consisted of two 3-min washes with water (5 mL of water per wash) to remove dust particles. The second cycle involved two 3-min washes with isopropanol (HPLC grade, Sigma-Aldrich) to remove potential lipophilic contaminants. Hair samples were then placed in Petri dishes and dried in the dark at room temperature for at least 48 h. Dried hairs were finely cut into 2–3 mm portions, and around 35 mg to 40 mg of each sample were weighed and placed in microtubes. To grind the hair samples, three stainless steel grinding balls were added to each tube, and the samples were briefly frozen by immersing the tubes in liquid nitrogen. The microtubes were then placed directly into a Retsch ball mill (MM 400, Verder Scientific, Eragny-sur-Oise, France) for two consecutive grinding cycles at 30 Hz for 10 min. A brief centrifugation was performed to gather the hair powder at the bottom of the tubes. To solubilize cortisol from the hair samples, 1.5 mL of methanol (HPLC grade, Sigma-Aldrich) was added to each tube. Extraction of cortisol from hair samples was carried out at room temperature for 24 h, with the tubes placed on a plate shaker set at 800 rpm. Then, the tubes were centrifuged at 1,500×*g* for 5 min, and a volume of 750 μL of supernatant was taken from each tube and transferred to new tubes. The new tubes were placed in a vacuum concentrator (5,303 vacuum concentrator, Eppendorf, Montesson, France) at 45°C for approximately 1 h to evaporate the methanol. Finally, tubes containing cortisol in solid form were stored at −20°C until further analysis. Cortisol was re-solubilized in 140 μL of PBS solution and measured in duplicate using an ELISA kit for free cortisol in saliva (Demeditec Diagnostics GmbH, Kiel, Germany) following the manufacturer’s instructions. Concentrations were converted from ng/mL to pg/mg of hair for data analysis. The detection limit of the ELISA kit was 0.019 ng/mL (i.e., 0.13–0.15 pg/mg for hair samples weighing between 35 and 40 mg).

### Recording of management practices and herd health data

Management practices and herd health data were recorded at the first visit. The same recording was repeated 8 months later, at the second visit, to identify any changes in management practices or the occurrence of health events between the two visits. Data requested from farmers included: (1) the procedures applied to piglets in the farrowing unit (castration, tail docking, teeth grinding, iron administration), (2) the age at which weaning and other social regrouping took place (i.e., when pigs were moved from one pen to another, resulting in a new social structure and potentially the establishment of a new social hierarchy) and the criteria used to distribute pigs in the new pens (i.e., litter, sow parity, weight or pig sex), (3) the minimal floor space allowance per pig in the post-weaning and finishing units. Housing facilities were described at each visit, indicating whether they were indoors, indoors with outdoors access, or outdoors. With regard to herd health, the herd veterinarian was asked to provide information on: (1) any health disorder requiring the formulation of a health plan on the farms recruited, (2) the physiological stages affected by these health disorders, and (3) whether there had been any change in herd health between the first and second visits. Change in herd health statuses was identified by veterinarians through clinical observations of animals. All data are displayed in Supplementary Tables S1, S2.

### Data analyses

Statistical analyses were carried out using R version 4.0.5. No transformation or adjustment were made to the raw data. No outlier was identified in the raw data.

Firstly, the distribution of cortisol concentration was described for the entire population of sampled pigs and separately for each farm’s batch.

Secondly, to characterize the variability in hair cortisol concentration based on farm and batch, an analysis of variance model was implemented using the *aov* function (*stats* package). The model was validated by an analysis of residuals. Normality was assessed using a graphical analysis (QQ-plot) and a Shapiro–Wilk test, while homoscedasticity was assessed using a graphical examination of residual linearity and a Levene test. The analysis of variance model included the effects of batch, farm, and their interaction. Variables were considered significant if *p*-values were < 0.05 (*anova* test). To determine the proportion of variance explained by each predictor variable (batch, farm and their interaction), the effect size η^2^ was calculated (ratio of the sum of squares of a predictor variable to the total sum of squares of the model). The η^2^ indicator describes the proportion of concentration variability explained by each variable, after adjustment for other predictor variables. A pairwise *post-hoc* test (Tukey’s test) was performed to compare mean hair cortisol concentrations between batches from the same farm. The absolute value of Cohen’s *d* was calculated for comparisons of means between the two batches on all farms (ratio between the difference of the means of compared batches and the standard deviation) (30). Cohen’s *d* is a complementary indicator to the *p*-value, providing an estimate of the magnitude of the difference between two means.

Thirdly, to determine the presence of farm profiles, a Principal Component Analysis (PCA) followed by a Hierarchical Clustering on Principal Components (HCPC) was carried out. Farms were distinguished using four active variables (i.e., variables contributing to the distinction of farms). The active variables represented the means and standard deviations of hair cortisol concentration obtained per farm, considering both batches. Seventeen supplementary variables were considered (i.e., they do not contribute to the distinction of farms but provide information on the characteristics of the farm clusters identified). The supplementary variables included management practices and pig health data, as reported by farmers and veterinarians. They included: (1) castration, (2) tail docking, (3) teeth grinding, (4) iron administration, age at potential social regroupings, (5) during the suckling period (i.e., early socialization), (6) at weaning, (7) during fattening, and their (8–10) respective modalities (i.e., regrouping based on litter, sow parity, weight or pig sex), type of housing facilities (i.e., indoors, indoors with outdoors access, outdoors) during the (11) suckling, (12) post-weaning and (13) fattening periods, the minimal floor space allowance per pig in the (14) post-weaning and (15) fattening units, (16–17) the presence of a health disorder for each batch.

Finally, a sampling procedure was implemented to determine how the mean and standard deviation of hair cortisol concentration in a batch varied with decreasing sample size. For all batches, all the possible samples of 5, 10, 15, and 20 pigs were drawn from the initial sample of 24 animals. Two calculations were performed for each sample: (1) the relative difference between the means of the drawn samples and the initial sample (i.e., with the 24 sampled animals; Equation 1), and (2) the relative difference between the standard deviations of the drawn samples and the initial sample (Equation 2). The distributions of the relative differences in means and standard deviations were described. Summary statistics (5th, 50th, and 95th percentiles) were computed for the relative differences in means and standard deviations for all sample sizes (5, 10, 15, and 20 pigs) in all batches studied (40 batches). Forty percentile values were obtained for each sample size. The means of the summary statistics (5th, 50th, and 95th percentiles) were computed for each sample size (5, 10, 15, and 20 pigs) in all batches.


(1) $$\text{Relative}\;\text{difference}\;\text{between}\;\text{means}\;\left(\%\right):\Delta_{i,j,n}=\frac{\mu_{i,j}-\mu_{n}\;}{\mu_{n}}$$

(2) $$\begin{array}{c} \text{Relative}\;\text{difference}\;\text{between}\;\text{standard}\;\text{deviations}\;\left(\%\right):\\ \Delta \prime_{i,j,n}=\frac{\sigma_{i,j}-\sigma_{n}}{\sigma_{n}} \end{array}$$

Where μ = mean; σ = standard deviation; i = number of the drawn sample, i ϵ [1; N]; j = size of the drawn sample, j = {5; 10; 15; 20}; n = identification number of a given farm, n ϵ [1; 20].

## Results

### Interindividual variability in hair cortisol concentration at the sampled population level

**Figure 1:**
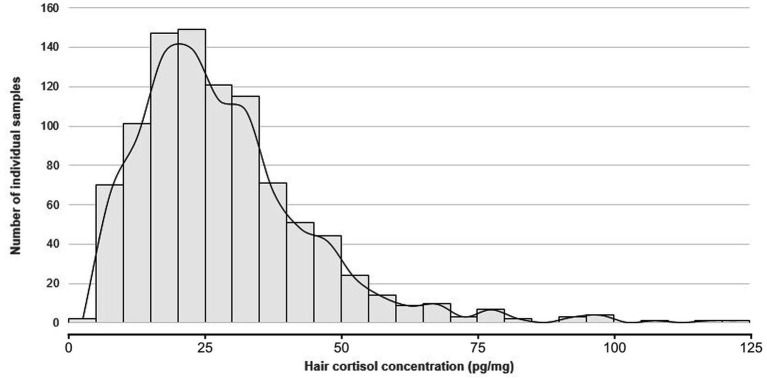
Distribution of hair cortisol concentrations in 950 finishing pigs sampled in two batches per farm on 20 farms.

The distribution of hair cortisol concentration of 950 pigs raised on 20 farms (two batches per farm) is shown in Figure 1. Of note, 10 pigs from five batches on five farms were not sampled due to premature departure for slaughter. The distribution appears to be asymmetrical. Hair cortisol concentrations ranged from 0.4 to 121.6 pg/mg, with 50% of the values <25 pg/mg. The mean hair cortisol concentration was 25.9 pg/mg (standard deviation = 16.2 pg/mg).

### Interindividual variability in hair cortisol concentration within a batch

**Figure 2:**
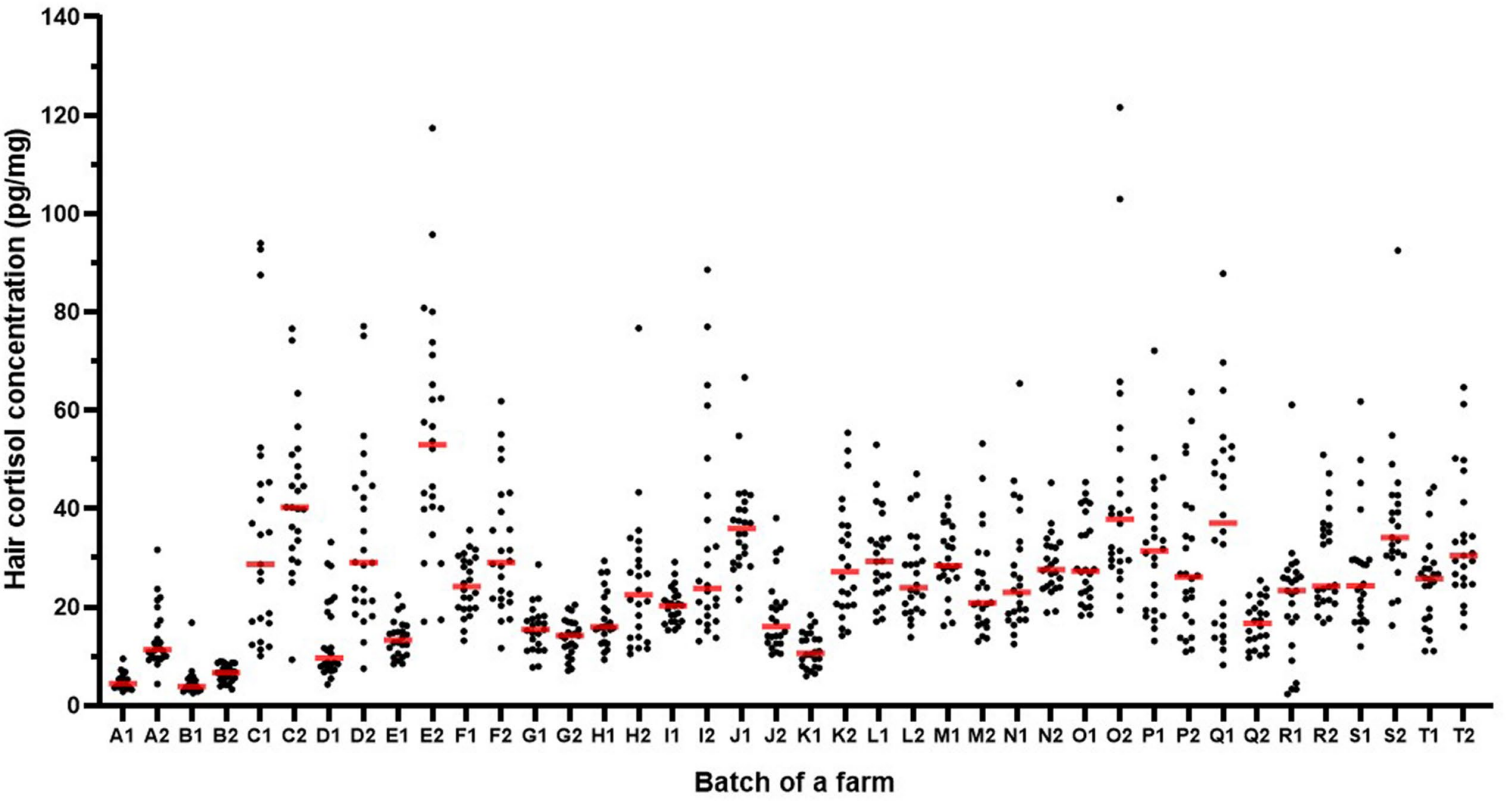
Hair cortisol concentration in finishing pigs sampled in two batches per farm (1 and 2) on 20 farms (A to T). Each dot corresponds to the concentration of one pig and the red lines indicate the median concentration of a batch.

**Figure 3:**
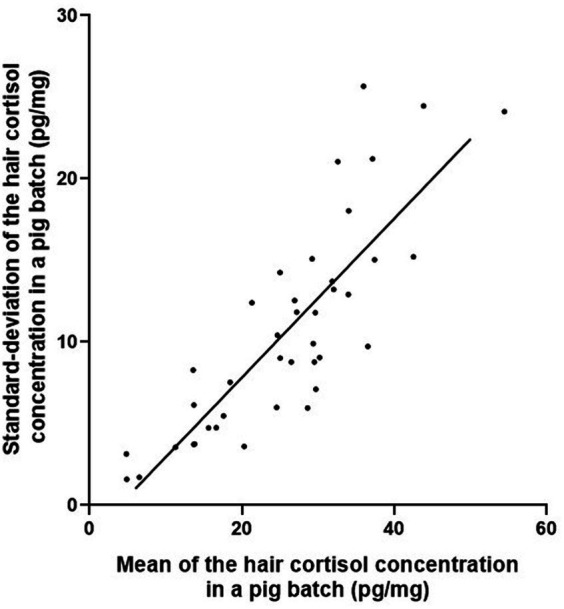
Linear regression of standard deviation against mean hair cortisol concentration in finishing pigs sampled in two batches per farm on 20 farms (one dot corresponds to one batch).

The hair cortisol concentrations in pigs sampled in the two batches 8 months apart are shown farm by farm in Figure 2. The coefficients of variation for hair cortisol concentration within a batch ranged from 0.17 to 0.71. The standard deviation of hair cortisol concentration within a batch increased as the mean hair cortisol concentration of the batch increased (correlation coefficient: 0.83; *p* < 0.001; Figure 3). No batch had a high mean and a low standard deviation of hair cortisol concentration. For mean values ranging from 20 to 50 pg/mg, some batches showed higher homogeneity while others displayed higher heterogeneity (coefficient of variation ranging from 0.18 to 0.64).

The proportion of variability (η^2^) explained by the three predictor variables in the analysis of the variance model (batch, farm and their interaction) is presented in Table 1. The residuals explained 57% of the total variability in hair cortisol concentration (Table 1). Residuals represent the portion of the variability not explained by the predictor variables in the analysis of the variance model (batch, farm and their interaction), thus corresponding to interindividual differences.

**Table 1:** Proportion of the variability in hair cortisol concentration of finishing pigs sampled from two batches per farm on 20 farms, as a function of the predictor variables included in the analysis of variance model.

Predictor variable	Percentage of variance explained (η^2^)
Batch	3.1
Farm	24.1
Batch: Farm	16.3
Residuals	56.5
Total	100

### Variability in hair cortisol concentration between farms

The farm effect explained 24% of the total variability in hair cortisol concentration (without considering its interaction with the batch effect, Table 1). Farms A, B, and G had the lowest hair cortisol concentration (Table 2).

**Table 2:** Hair cortisol concentrations (pg/mg) in two batches of finishing pigs sampled 8 months apart on 20 farms (24 pigs were sampled per batch regardless of the total number of pigs in the batch).

	First batch		Second batch			
Farm	Mean (μ1)	Standard deviation	Mean (μ2)	Standard deviation	Cohen’s *d*^1^ (absolute value)	*p*-value (Tukey’s test)
A	4.9	1.6	13.7	6.1	1.4	ns
B	4.8	3.1	6.5	1.7	0.7	ns
C	35.9	25.7	42.5	15.2	0.3	ns
D	13.6	8.3	34.0	18.0	1.2	<0.001
E	13.7	3.7	54.5	24.1	1.5	<0.001
F	24.5	6.0	32.0	13.2	0.7	ns
G	15.6	4.7	13.8	3.7	0.4	ns
H	17.6	5.5	25.0	14.3	0.7	ns
I	20.3	3.6	32.6	21.1	0.8	ns
J	36.5	9.7	18.4	7.5	1.4	<0.001
K	11.3	3.6	29.6	11.8	1.4	<0.001
L	30.2	9.1	26.5	8.8	0.4	ns
M	29.7	7.1	24.6	10.4	0.6	ns
N	26.9	12.6	28.6	6.0	0.2	ns
O	29.5	8.8	43.8	24.5	0.7	<0.05
P	31.8	13.7	29.2	15.1	0.2	ns
Q	37.1	21.2	16.6	4.8	1.1	<0.001
R	21.3	12.4	29.4	9.9	0.7	ns
S	27.2	11.8	37.4	15.0	0.7	ns
T	25.0	9.0	33.9	12.9	0.6	ns

^1^Cohen’s *d* is an indicator to estimate the magnitude of the difference between two means (expressed as a ratio between the difference of the two means and the standard deviation).

### Variability in hair cortisol concentration between batches

The mean hair cortisol concentration ranged from 4.8 to 54.5 pg/mg across batches. The batch effect, without considering its interaction with the farm effect, explained the lowest proportion of the variability in hair cortisol concentration (η^2^ = 3%, Table 1). The batch:farm interaction effect explained 16% of the total variability in hair cortisol concentration (Table 1).

Within a given farm, the mean hair cortisol concentration in batch 1 could be significantly lower (on 4 farms), higher (on 2 farms), or not significantly different (on 14 farms) from that in batch 2 (Table 2). All Cohen’s *d* values were > 1 on farms where the mean hair cortisol concentration differed significantly between the two batches, except for farm O (indicating a difference of more than one standard deviation between the two compared means). Farm A was the only one with a Cohen’s *d* > 1.0 and a non-significant difference in mean concentrations between the two batches (*p* > 0.05, Tukey’s test).

On the two farms where the mean hair cortisol concentration in batch 1 was significantly higher than that in batch 2, veterinarians reported an improvement in health (farms J, Q). Also, castration was ceased on farm Q, while no change in management practices was noted on farm J between the two samplings. Among the four farms where the mean hair cortisol concentration in batch 1 was significantly lower than that in batch 2, veterinarians observed: an improvement in health between the two batches sampled 8 months apart on farm D; the occurrence of a health disorder in the second batch on farm O; the presence of an unchanged severity of a health disorder on farm K; and no health disorder on farm E. No change in management practices was reported on these four farms between the two batches (farms D, E, K, O).

### Clustering of farms based on hair cortisol concentration

The first two principal dimensions of the PCA explained 91.5% of the variance (Supplementary Figure S1). HCPC allowed the identification of three clusters of farms, denoted as C1 (three farms), C2 (seven farms), and C3 (10 farms). The *v.test* value is provided in Table 3 when P was <0.05.

**Table 3:** Characterization of farm clusters identified by hierarchical clustering on the four active variables and the 17 supplementary variables.

	Cluster		
	C1	C2	C3
Number of farms in the cluster	3	7	10
Value of *v.test* of active variables			
Mean of the hair cortisol concentration in pigs in batch 1	−2.73		3.29
Mean of the hair cortisol concentration in pigs in batch 2	−2.85	2.10	
Standard deviation of the hair cortisol concentration in pigs in batch 1			3.07
Standard deviation of the hair cortisol concentration in pigs in batch 2	−2.43	3.02	
Value of *v.test* of supplementary variables			
Tail docking (no)	2.41		
Early socialization in the suckling period (no)	−1.99		
Age at weaning (42-day-old)	2.41		
Regrouping in fattening units (weight)		2.68	
Housing facilities during the post-weaning period (closed with outdoor facilities)	2.41		
Housing facilities during the fattening period (closed with outdoor facilities)	2.41		
Minimal floor space allowance per pig during post-weaning period (<0.3 m^2^/porc)	−1.99		
Minimal floor space allowance per pig during fattening period (>1.5 m^2^/porc)	2.41		

The *v.test* value is indicated when *P* < 0.05. A positive (or negative) *v.test* value for quantitative variables indicates a higher (or lower) mean or standard deviation in the cluster compared to the cohort. A positive (or negative) *v.test* value for qualitative variables indicates overrepresentation (or underrepresentation) of a modality in the cluster compared to the cohort. The higher the absolute value of the *v.test*, the more the group is characterized by this variable (or modality). Only supplementary variables for which at least one modality had a *p* < 0.05 are shown in the table (modality indicated in brackets). C1, Group of farms with lower means of hair cortisol concentration for both batches and lower interindividual differences only for batch 2, compared to the cohort. C2, Group of farms with higher mean and interindividual differences of hair cortisol concentration in batch 2, compared to the cohort. C3, Group of farms with higher mean and interindividual differences of hair cortisol concentration in batch 1, compared to the cohort.

#### Cluster 1 (A, B, G)

This cluster was defined by lower means of hair cortisol concentration than those of the cohort for both batches, and a lower intra-batch variability for batch 2 only. This cluster was characterized by seven supplementary variables, with the following modalities (Table 3): no tail docking, early socialization in the farrowing unit, weaning age of 42-day-old, post-weaning and finishing housing facilities providing outdoors access, a minimal floor space allowance per pig, respectively, not <0.3 m^2^ and > 1.5 m^2^ in the post-weaning and the finishing pens. These characteristics corresponded to farms A and B, which were the only two organic farms in the cohort. Farm G, a conventional farm without specification, had very different management practices. Indeed, pigs on farm G were tail docked, not early socialized in the farrowing unit, weaned at 28-day-old, raised in post-weaning and finishing facilities without outdoors access, with a minimal floor space allowance per pig <0.3 m^2^/pig in post-weaning and 0.7–0.9 m^2^/pig in finishing (Supplementary Tables S1, S2).

#### Cluster 2 (D, E, F, H, I, K, O)

This cluster was defined by higher means and intra-batch variabilities of hair cortisol concentration in batch 2 compared to the cohort. The mean hair cortisol concentration was significantly different between the two batches on four farms out of seven (farms D, E, K, O). This cluster was characterized by one supplementary variable with the following modality (Table 3): the composition of pig pens in the finishing stage was based on the weight of pigs at the end of the post-weaning period, resulting in new groups of pigs and novel social interactions among pigs.

#### Cluster 3 (C, J, L, M, N, P, Q, R, S, T)

This cluster was defined by higher means and intra-batch variabilities of hair cortisol concentration in batch 1 compared to the cohort. The mean hair cortisol concentration was similar and high for the two batches in this cluster, except for farms J and Q. No supplementary variable was associated with this cluster.

### Sample size and variability in hair cortisol concentration

**Figure 4:**
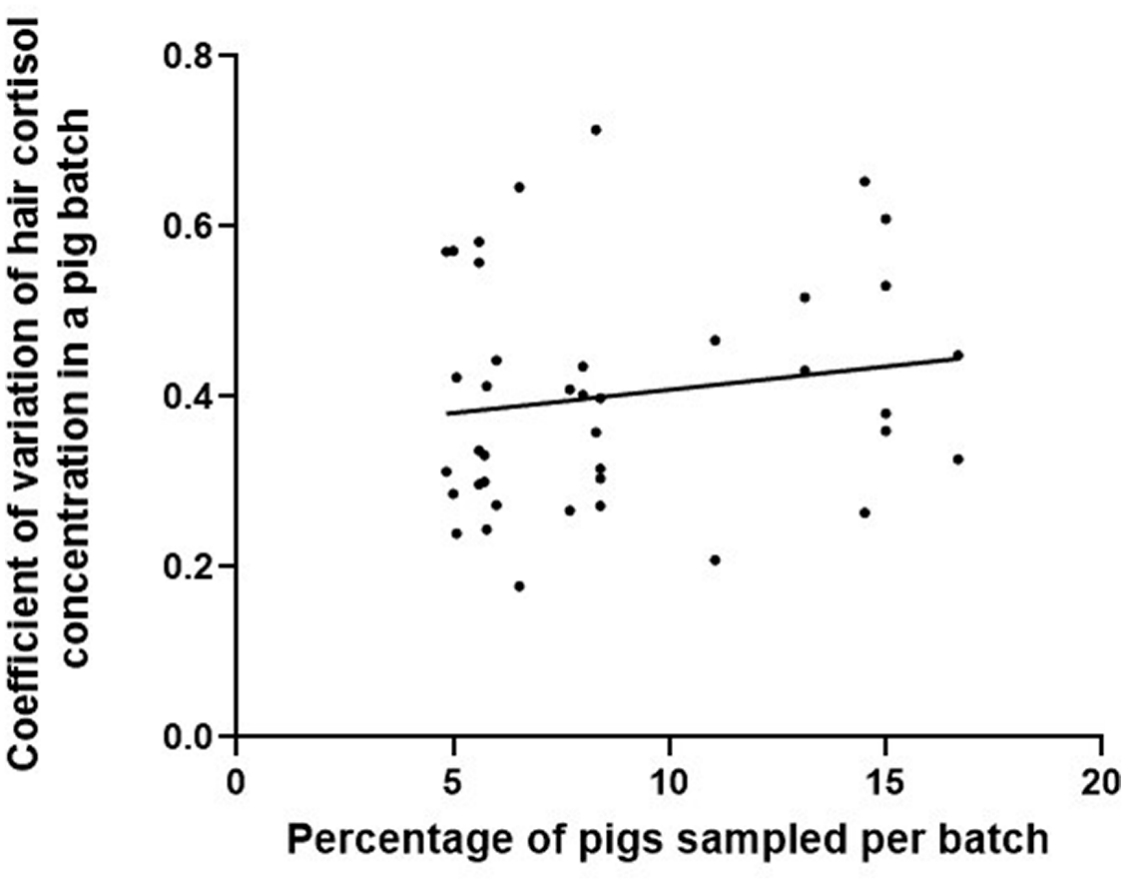
Linear regression of the coefficient of variation of hair cortisol concentrations against the percentage of finishing pigs sampled in two batches per farm on 20 farms (one dot corresponds to one batch).

The percentage of pigs sampled per batch ranged from about 5 to 17% of the total number of finishing pigs, with a median percentage around 8%. The coefficient of variation of hair cortisol concentration per batch was not correlated with the percentage of pigs sampled per batch (correlation coefficient: 0.15; *p* = 0.33; Figure 4). For example, when considering a similar proportion of pigs sampled per batch (about 8%), the coefficient of variation ranged from 0.18 to 0.71. The farms with the lowest and highest proportion of pigs sampled per batch in the cohort showed a high and a very low intra-batch variability, respectively. The farm with the lowest percentage of pigs sampled per batch in the cohort (farm H, 5%) had a standard deviation of hair cortisol concentration higher than the third quartile in batch 2 compared to the cohort (14.3 pg/mg). The farm with the highest percentage of pigs sampled per batch in the cohort (farm A, 17%) had the lowest standard deviation of hair cortisol concentration (1.6 pg/mg, batch 1).

**Figure 5:**
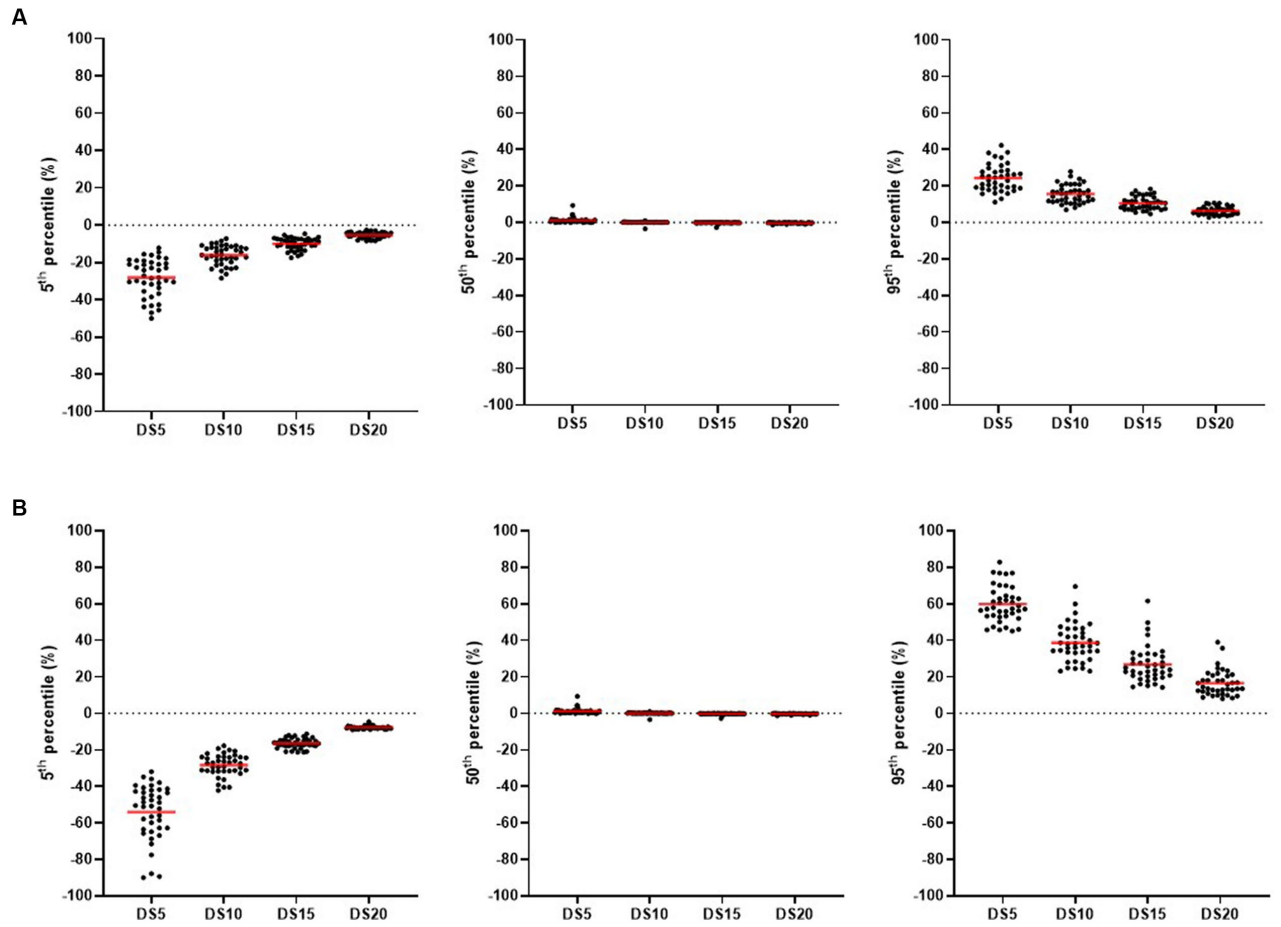
Percentiles (5th, 50th, 95th) of relative differences between the means **(A)** or standard deviations **(B)** in hair cortisol concentration in the initial sample and drawn sample (DS) of n pigs (DS5: *n* = 5 pigs; DS10: *n* = 10 pigs; DS15: *n* = 15 pigs; DS20: *n* = 20 pigs). In all batches of finishing pigs (two batches per farm on 20 farms), all possible samples of 5, 10, 15, and 20 pigs were drawn from the initial sample of 24 pigs. For each drawn sample, relative differences were calculated between the means and standard deviations of the initial sample and the random sample. For each batch (*n* = 40), summary statistics (5th, 50th, 95th percentiles) of these relative differences were computed for all the drawn samples (one dot corresponds to the percentile value of one batch, for a given sample size). The mean of a summary statistic for one size of drawn sample is indicated by a red line.

Figure 5 shows the relative differences from the initial means (Figure 5A) and initial standard deviations (Figure 5B), according to the size of the drawn samples (5, 10, 15, or 20 pigs from the initial batch of 24 pigs). The relative differences were lower in means than in standard deviations, all sizes of the random samples considered. The relative difference in standard deviations was higher in batches where interindividual differences in hair cortisol concentration were large, even with drawn samples of 20 pigs.

## Discussion

This exploratory study is to our knowledge the first one to describe the variability in hair cortisol concentration in finishing pigs on several commercial farms. We aimed to characterize differences between pigs within a batch, between farms (which could reflect different levels of exposure to stressors), and between batches within the same farm. The secondary aim was to determine how the number of sampled pigs influences the characterization of hair cortisol concentration within a batch.

### Variability in hair cortisol concentration explained by interindividual differences in stress response

Interindividual differences explained more than half of the variability in hair cortisol concentration. The range of reported hair cortisol concentration for a total of 421 finishing pigs in six previous studies (ranging from 4.5 to 177.6 pg/mg) is similar to the one reported in this study (with 2.3 times more sampled pigs; ranging from 0.4 to 121.6 pg/mg), except for the lowest values (19–23, 26). The lowest hair cortisol concentration measured in this study had not yet been described in finishing pigs but had been reported in sows (31). Of note, no study comparing inter-laboratory assay methods to measure hair cortisol concentration was, to our knowledge, published. To validate the comparability between hair cortisol concentration measured on different laboratories, further comparison should be carried out.

Differences between pigs of a same batch in hair cortisol concentration could potentially be explained by different stress responses to the expositions of same stressors. For instance, Casal et al. (23) demonstrated that hair cortisol concentration in 21-week-old pigs, subjected to repeated regroupings once a week for the 4 weeks preceding sampling, could differ among individuals (with minimum and maximum concentrations of 6.4 and 40.0 pg/mg). Differences in hair cortisol concentration between pigs after repeated regroupings could be explained by differences in their individual characteristics, such as differences in their dominance ranks (32). Nonetheless, this study was not designed to describe different stress response between pigs for the same exposure to a stressor. Reasons explaining the concentration variability between finishing pigs within the same batch should be explored to better understand the information provided by hair cortisol at the pig level.

### Variability in hair cortisol concentration explained by differences between farms

Differences between farms explained nearly a quarter of the variability in hair cortisol concentration. To our knowledge, this is the first description of differences in hair cortisol concentration at the end of the finishing period between commercial farms. It is likely that the diverse management practices and health disorders in recruited pig farms contribute to the differences between farms. These farm-level differences suggest the need for further investigations into the factors influencing hair cortisol concentration throughout the lifetime of pigs in farming systems.

The clustering revealed that a minority of farms had low means and differences between individuals in hair cortisol concentration whatever the batch. The two organic farms of the cohort were included in this cluster. These results are interesting because organic management practices, such as early socialization, weaning at 6 weeks of age, and outdoors access, are generally perceived to provide better animal welfare than conventional management practices (33, 34). Moreover, all growing pigs within batches of these two organic farms were housed in one or two pens since weaning. Social stress in these farms could be limited since social hierarchy was early established and not further modified. However, it is not possible to conclude from this study that organic management practices are less stressful for pigs than the other types of management practices. Indeed, only two organic farms were monitored. Moreover, the study design was not suitable for assessing the association between hair cortisol concentration and management practices. Furthermore, the other farm included in the same cluster than the two organic farms was conventional and without specification. We can only suppose that the pigs having the lower hair cortisol concentrations were less exposed to stressors than the others. We hypothesize that a low exposure to stressors limits the observations of large interindividual differences within a batch.

### Variability in hair cortisol concentration explained by differences between batches

This study is, to our knowledge, the first to evidence differences in hair cortisol concentration between two batches within the same farm. These differences imply that hair should be sampled in more than one batch on farm, if the perspective of using hair cortisol is to assess long-term stress of pigs at the farm level. The previously reported mean or median values of a batch (ranging from 4.5 to 54.3 pg/mg; 19–23.26), for pigs sampled at the end of the finishing period (between 20 and 40 weeks of age), were comparable to the mean batch values obtained in this field study (ranging from 4.8 to 54.5 pg/mg). Differences in hair cortisol concentration between two batches within the same farm could potentially be attributed to changes in the nature or intensity of stressors. Hypotheses to further investigate could include changes in management practices, social stress due to pig behavior, thermal stress or health events (22, 35–39).

### Sample size for observing variability in hair cortisol concentration

The low interindividual differences in a few batches do not appear to be due to sampling bias resulting from a small number of sampled pigs. Indeed, the farm with the lowest percentage of pigs sampled per batch had high interindividual differences. Inversely, the farm with the highest percentage of pigs sampled per batch showed very low interindividual differences. So, the variability in concentration within a batch seems to be a characteristic of this batch.

Characterizing the hair cortisol concentration of a batch requires an adequate sample size to calculate both the mean and the standard deviation. While a sample size of 15 pigs per batch can be sufficient to estimate the mean hair cortisol concentration in a batch, more than 24 pigs seem to be needed to better describe the variability between pigs. Indeed, the mean value of hair cortisol concentration in a drawn sample of 15 pigs differed relatively little from the initial sample of 24 pigs, resulting in a similar classification as low, intermediate, or high mean concentration. However, the differences in described variability between samples of 20 and 24 pigs suggest that there is still room for more precision in describing the variability with a larger sample size. That is why more than 24 pigs seem to be needed to better describe the variability between pigs.

## Conclusion

The hair cortisol concentration in finishing pigs on commercial farms showed large differences between pigs within a batch, between farms and to a lesser extent between batches of the same farm. The described variability suggests that there is an interest in further investigating if hair cortisol can be a biomarker to assess long-term stress in pigs on commercial farms. The differences between farms and batches confirm the need to assess whether it can be linked to long-term exposure of pigs to stressors. Insights about the sampling of finishing pigs were obtained in this study. More than 24 finishing pigs per batch are needed to describe the variability between pigs within a batch whereas 15 pigs could be sufficient to estimate the mean hair cortisol concentration of the batch. More than one batch is needed to describe the hair cortisol concentration at the farm level.

## Data availability statement

The datasets presented in this study can be found in an online repository. The names of the repository and accession number can be found at: https://zenodo.org/record/8359352 (40).

## Ethics statement

Ethical approval was not required for the study involving animals in accordance with the local legislation and institutional requirements because no ethical approval was required for this study, since the sampling of pig hair was carried out on commercial farms. According to French regulation (Rural and Sea fishing Code, book II, first title, chapter IV, section 6), ethic committees only provide authorizations for research conducted under experimental conditions. Hair sampling was non-invasive and was carried out with the approval of farmers.

## Author contributions

PL: Data curation, Formal analysis, Investigation, Validation, Visualization, Writing – original draft. ML-M: Conceptualization, Investigation, Supervision, Validation, Writing – review & editing. AL: Data curation, Formal analysis, Validation, Writing – review & editing. SG: Resources, Validation, Writing – review & editing. BL: Validation, Writing – review & editing. JH: Validation, Writing – review & editing. CF: Conceptualization, Funding acquisition, Supervision, Validation, Writing – review & editing. CB: Conceptualization, Funding acquisition, Investigation, Supervision, Validation, Writing – review & editing.
