# Supplementary material for: Hair cortisol concentration in finishing pigs on commercial farms: variability between pigs, batches, and farms
Source: Front Vet Sci. 2024 Jan 22;10:1298756. doi: 10.3389/fvets.2023.1298756 (PMC10839108; doi:10.3389/fvets.2023.1298756)
Supplement: Supplementary file 1 [file Data_Sheet_1.docx]

Supplementary Material

Hair cortisol concentration in finishing pigs on commercial farms: variability between pigs, batches and farms

**Pierre Levallois^1^, Mily Leblanc-Maridor^1^, Anne Lehébel^1^, Solenn Gavaud^2^, Blandine Lieubeau^2^, Julie Hervé^2^, Christine Fourichon^1^, Catherine Belloc^1*^**

^1^Oniris, INRAE, BIOEPAR, 44300 Nantes, France

^2^Oniris, INRAE, IECM, 44300 Nantes, France

*** Correspondence:** catherine.belloc@oniris-nantes.fr


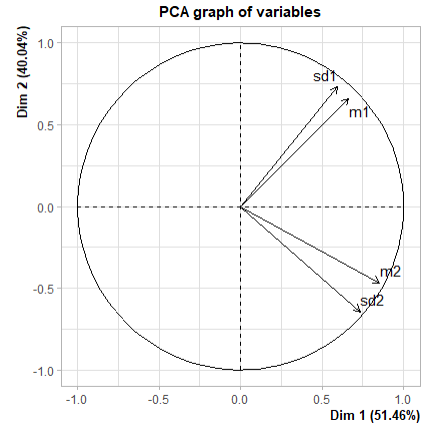


**Figure S1:** Graph of the variables obtained from the Principal Components Analysis (R version 4.0.5). The four active variables were the means (m1, m2) and the standard deviations (sd1, sd2) in hair cortisol concentration in the finishing pigs in two batches on 20 farms.

**Table S1.** Description of the management practices and social regrouping on the 20 farms that could induce differences in exposure of pigs to stressors during their lifetime on the farm.

| **Farm** | **Cluster** | **Management practices** | | | | | **Social regrouping (*i.e. when pigs are moved from one pen to another involving a new social structure and possibly the establishment of a new social hierarchy*)** | | | | | |
| --- | --- | --- | --- | --- | --- | --- | --- | --- | --- | --- | --- | --- |
|  |  | **Specification** | **Castration** | **Tail docking** | **Teeth grinding** | **Iron administration** | **Age at a new social regrouping (days)** | | | **Type of social regrouping** | | |
|  |  |  |  |  |  |  | **Suckling** | **Weaning** | **Fattening** | **Suckling** | **Weaning** | **Fattening** |
| **A** | 1 | Organic | Yes | No | No | No | 1 | 42 | /^1^ | All pens are connected | All litters are regrouped in two pens | / |
| **B** | 1 | Organic | No | No | No | Oral | 21 | 42 | / | Two pens are connected | All litters are regrouped in one pen | / |
| **C** | 3 | Antibiotic-free from 42-day-old | No | Yes | Yes | IM^4^ | / | 28 | 84 | / | Weight^2^ | Pen divided into two^3^ |
| **D** | 2 | None | Yes | Yes | No | IM | / | 28 | 77 | / | Litters^5^ | Pen divided into two |
| **E** | 2 | None | Yes | Yes | Yes | Oral | / | 21 | 60 | / | Weight | Weight |
| **F** | 2 | Label Rouge | Yes | Yes | No | IM | / | 28 | 84 | / | Weight | Weight |
| **G** | 1 | None | Yes | Yes | Yes | IM | / | 28 | 70 | / | Litters | Ramdomly^6^ |
| **H** | 2 | Label Rouge | Yes | Yes | No | IM | / | 21 | 77 | / | Weight | Weight |
| **I** | 2 | None | Yes | Yes | No | IM | / | 28 | 75 | / | Weight | Weight |
| **J** | 3 | None | Yes | Yes | Yes | Oral | / | 21 | 77 | / | Litters | Weight |
| **K** | 2 | Label Rouge | Yes | Yes | Yes | IM | / | 28 | 75 | / | Litters | Weight |
| **L** | 3 | None | Yes | Yes | Yes | IM | / | 28 | / | / | Parity^7^ and weight | / |
| **M** | 3 | None | Yes | Yes | No | Oral | / | 28 | 77 | / | Weight | Pen divided into two |
| **N** | 3 | None | Yes | Yes | No | Oral | / | 25 | 75 | / | Parity and litters | Pen divided into two |
| **O** | 2 | Antibiotic-free from birth | No | Yes | No | IM | / | 28 | / | / | Parity and weight | / |
| **P** | 3 | None | No | Yes | Yes | Oral | / | 28 | 77 | / | Weight | Pen divided into two |
| **Q** | 3 | None | Yes then no^8^ | Yes | Yes | IM | / | 21 | 77 | / | Weight | Randomly |
| **R** | 3 | Antibiotic-free from birth | No | Yes | Yes | Oral | / | 28 | / | / | Weight | / |
| **S** | 3 | Label Rouge | Yes | Yes | Yes | IM | / | 28 | 84 | / | Parity and weight | Sex^9^ and weight |
| **T** | 3 | None | Yes | Yes | No | IM | 15 | 28 | / | Two pens are connected | Weight and litters | / |

1. / = Not concerned.
2. Weight = Animals are regrouped in a new pen according to their weight. Animals in the same pen have thus a homogeneous weight and heterogeneous origin.
3. Pen divided into two = Animals in one pen of the former unit (*e.g.* post-weaning unit) are allocated to two pens in the next unit (*e.g.* fattening unit).
4. IM = IntraMuscular injection.
5. Litters = Piglets are regrouped in post-weaning pens according to their litters, without considering the parity of the sows. Usually, two litters constituted one pen.
6. Randomly = Animals are regrouped in a new pen without considering their former litter, their weight, their sex or sow parity. Usually, animals from three to five pens were moved together and randomly allocated to new pens.
7. Parity = Piglets are regrouped according to sow parity. Attention was paid particularly to piglets born from sows having a parity lower than 3. Exceptions could be made when parity was higher than three: piglets born from sows having different parity higher than three could be mixed.
8. Yes then no = Pigs sampled in the first batch were castrated whereas those sampled in the second batch were not.
9. Sex = Pigs were regrouped according to their sex: males and females were not mixed in the same pen.

**Table S2.**Description of the housing facilities and health on the 20 farms that could induce differences in exposure of pigs to stressors during their lifetime on the farm.

| **Farm** | **Cluster** | **Housing facilities** | | | **Health** | | | |
| --- | --- | --- | --- | --- | --- | --- | --- | --- |
|  |  | **Thermal stress that could be induced by housing facilities** | **Minimal floor space allowance** **per pig in pens (m^2^/pig)** | | **Health disorder** | **Stage concerned by a health disorder** | **Evolution of a health disorder according to veterinarians** | **Batch concerned by this health disorder** |
|  |  |  | **Post-weaning** | **Fattening** |  |  |  |  |
| **A** | 1 | Outdoors at farrowing. Indoors with outdoors access from weaning to finish | 0.6-0.8 | >1.5 | Cough | Post-weaning | Improvement | 1 |
| **B** | 1 | Indoors with outdoors access from weaning to finish | >1.5 | >1.5 | /^1^ | / | / | / |
| **C** | 3 | Indoors. Housing facilities not entirely isolated in post-weaning units | <0.3 | 0.7-0.9 | Cough | Post-weaning | Deterioration | 1, 2 |
| **D** | 2 | / | <0.3 | 0.7-0.9 | Diarrhea related to  *L. intracellularis* | Fattening | Improvement | 1 |
| **E** | 2 | / | <0.3 | 0.7-0.9 | / | / | / | / |
| **F** | 2 | / | <0.3 | 1.2-1.5 | Diarrhea | Suckling | Deterioration | 1, 2 |
| **G** | 1 | / | <0.3 | 0.7-0.9 | / | / | / | / |
| **H** | 2 | / | <0.3 | 1.2-1.5 | Diarrhea | Suckling | Improvement | 1 |
| **I** | 2 | / | <0.3 | 0.7-0.9 | Nervous related to *S. suis* | Post-weaning | Improvement | 1 |
| **J** | 3 | / | <0.3 | 0.7-0.9 | PRRS | Fattening | Improvement | 1 |
| **K** | 2 | / | <0.3 | 1.2-1.5 | Diarrhea related to  *L. intracellularis* | Fattening | No evolution | 1, 2 |
| **L** | 3 | / | <0.3 | 0.7-0.9 | / | / | / | / |
| **M** | 3 | / | <0.3 | 0.7-0.9 | / | / | / | / |
| **N** | 3 | / | 0.3-0.5 | 0.7-0.9 | Tail biting | Post-weaning and fattening | Deterioration | 1, 2 |
| **O** | 2 | / | <0.3 | 0.7-0.9 | Cough related to  *M. hyopneumoniae* | Post-weaning | Improvement | 2 |
| **P** | 3 | / | <0.3 | 0.7-0.9 | Diarrhoea related to *E. coli* | Post-weaning | Deterioration | 1, 2 |
| **Q** | 3 | / | <0.3 | 0.7-0.9 | Diarrhea | Suckling | Improvement | 1 |
| **R** | 3 | Indoors. Housing facilities with a broken system of air circulation in fattening units | <0.3 | 0.7-0.9 | Cough | Fattening | Improvement | 1, 2 |
| **S** | 3 | / | <0.3 | 1.2-1.5 | / | / | / | / |
| **T** | 3 | / | <0.3 | 0.7-0.9 | / | / | / | / |

1. / = Not concerned.
